# Breast Cancer Cells Induce Stromal Fibroblasts to Secrete ADAMTS1 for Cancer Invasion through an Epigenetic Change

**DOI:** 10.1371/journal.pone.0035128

**Published:** 2012-04-13

**Authors:** Shiaw-Wei Tyan, Chih-Hung Hsu, Kai-Lin Peng, Chun-Chin Chen, Wen-Hung Kuo, Eva Y.-H. P. Lee, Jin-Yuh Shew, King-Jen Chang, Li-Jung Juan, Wen-Hwa Lee

**Affiliations:** 1 Genomics Research Center, Academia Sinica, Taipei, Taiwan; 2 Institute of Biochemistry and Molecular Biology, National Yang-Ming University, Taipei, Taiwan; 3 Department of Surgery, National Taiwan University Hospital, Taipei, Taiwan; 4 Cheng Ching General Hospital, Taichung, Taiwan; 5 Department of Developmental and Cell Biology, University of California Irvine, Irvine, California, United States of America; 6 Department of Biological Chemistry, University of California Irvine, Irvine, California, United States of America; The University of Arizona, United States of America

## Abstract

Microenvironment plays an important role in cancer development. We have reported that the cancer-associated stromal cells exhibit phenotypic and functional changes compared to stromal cells neighboring to normal tissues. However, the molecular mechanisms as well as the maintenance of these changes remain elusive. Here we showed that through co-culture with breast cancer cells for at least three to four passages, breast normal tissue-associated fibroblasts (NAFs) gained persistent activity for promoting cancer cell invasion, partly *via* up-regulating ADAM metallopeptidase with thrombospondin type 1 motif, 1 (ADAMTS1). Furthermore, we demonstrated that the DNA methylation pattern in the ADAMTS1 promoter has no alteration. Instead, the loss of EZH2 binding to the ADAMTS1 promoter and the resulting decrease of promoter-associated histone H3K27 methylation may account for the up-regulation of ADAMTS1. Importantly, the lack of EZH2 binding and the H3K27 methylation on the ADAMTS1 promoter were sustained in cancer cell-precocultured NAFs after removal of cancer cells. These results suggest that cancer cells are capable of inducing stromal fibroblasts to secrete ADAMTS1 persistently for their invasion and the effect is epigenetically inheritable.

## Introduction

Cancer development not only depends on the accumulation of genetic mutations in cancer cells, but also the interaction between cancer cells and their surrounding stroma. The tumor stroma including extracellular matrix (ECM), endothelial cells, pericytes, inflammatory cells and fibroblasts has been shown to assist cancer progression [1], [2], [3]. The cancer-associated stromal cells exhibit phenotypic and functional changes, as well as alterations in gene expression, compared to stromal cells neighboring to normal tissues [4], [5]. However, the molecular basis for these changes remains elusive. Nor do we understand in details how stromal fibroblasts facilitate cancer cell function. Our previous study suggests that the hepatocyte growth factor (HGF) released by the breast normal tissue-associated fibroblasts (NAFs) co-cultured with breast cancer cells is one of the contributing factors [4]. We also demonstrated that the HGF secretion, once established, can be maintained in NAFs without the continued co-culture with cancer cells [4]. Whether the maintenance requires epigenetic mechanism is not known.

ADAMTS1 has been shown to degrade ECM proteins such as aggrecan, versican and nidogen-1/-2 [6], [7], [8]. It helps the shedding of the extracellular membrane-bound proteins including heparin-binding epidermal growth factor-like growth factor and amphiregulin to activate EGF receptors [9], [10]. In addition, it cleaves membrane-associated syndecan-4 to disrupt cell adhesion and facilitate cell migration [11]. ADAMTS1 expression in cancer cells is capable of increasing tumor growth and cancer cell metastasis [10], [12].

In this communication, we show that, in addition to producing HGF for cancer cell growth [4], NAFs co-cultured with breast cancer cells also secreted ADAMTS1 for cancer cell invasion. Most importantly, we found that the above phenomenon can be maintained through an epigenetic mechanism.

## Results

### Breast cancer cells stimulated co-cultured fibroblasts to promote cancer cell invasion

**Figure 1:**
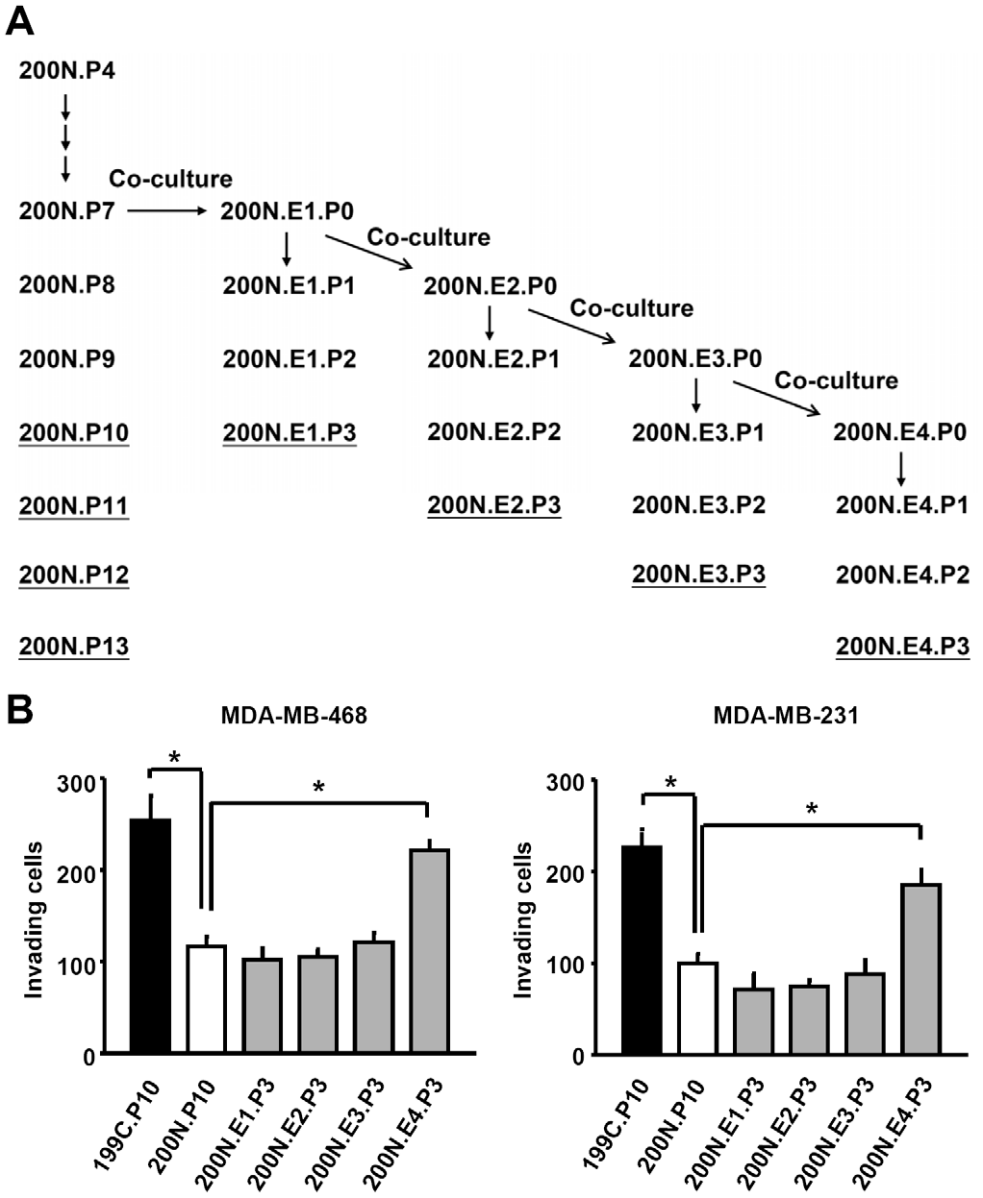
Pre-coculture with breast cancer cells enhanced fibroblast's ability to promote cancer cell invasion. (A) The diagram of the co-culture protocol. NAF 200N.P7 co-cultured with MDA-MB-468 cells for four passages is indicated as 200N.E1-E4, respectively. Each of 200N.E1-E4 was propagated in the absence of MDA-MB-468 cell for passages from P1 to P3. (B) The conditional media derived from CAF 199C.P10 and cancer cell-precocultured NAF 200N.E4.P3 enhanced the invasion ability of MDA-MB-468 cells and MDA-MB-231 cells. Data are shown as mean ± SD from triplicate experiments. Statistical significance was evaluated by Student's t-test. * *P*<0.05.

Our previous study has established a system in which NAF 200N was co-cultured with breast cancer MDA-MB-468 cells [4] (Fig. 1*A*). After co-incubation for 3.5 days, 200N cells were isolated and marked as 200N.E1.P0. Some of 200N.E1.P0 was propagated without MDA-MB-468 co-culture for further passages (P1, P2 and P3); others were continuously co-cultured with MDA-MB-468 to generate 200N.E2.P0, 200N.E3.P0 and 200N.E4.P0. These cells were again cultured with the removal of MDA-MB-468 for further passages P1, P2 and P3. 200N and its derivatives were then assayed for the ability in cancer cell invasion. The results demonstrated that the conditional medium derived from 200N.E4.P3 contained a higher activity in promoting invasion of MDA-MB-468 cells, compared to 200N (200N.P10) (Fig. 1*B*, left panel). Importantly, the invasion promoting capability of 200N.E4.P3 was comparable to that of cancer tissue-associated fibroblasts (CAF, 199C.P10). In contrast, 200N.E1-E3.P3 did not show increased activities for cell invasion (Fig. 1*B*, left panel). Similar results were observed when the other breast cancer MDA-MB-231 cells were used for invasion assay (Fig. 1*B*, right panel). These experiments indicate that cancer cells are able to stimulate normal tissue-associated fibroblasts to facilitate cell invasion, and that the effect requires a certain period of time of NAF co-culture with cancer cells before it can be established.

### Breast cancer cells induced ADAMTS1 expression in the co-cultured fibroblasts

**Figure 2:**
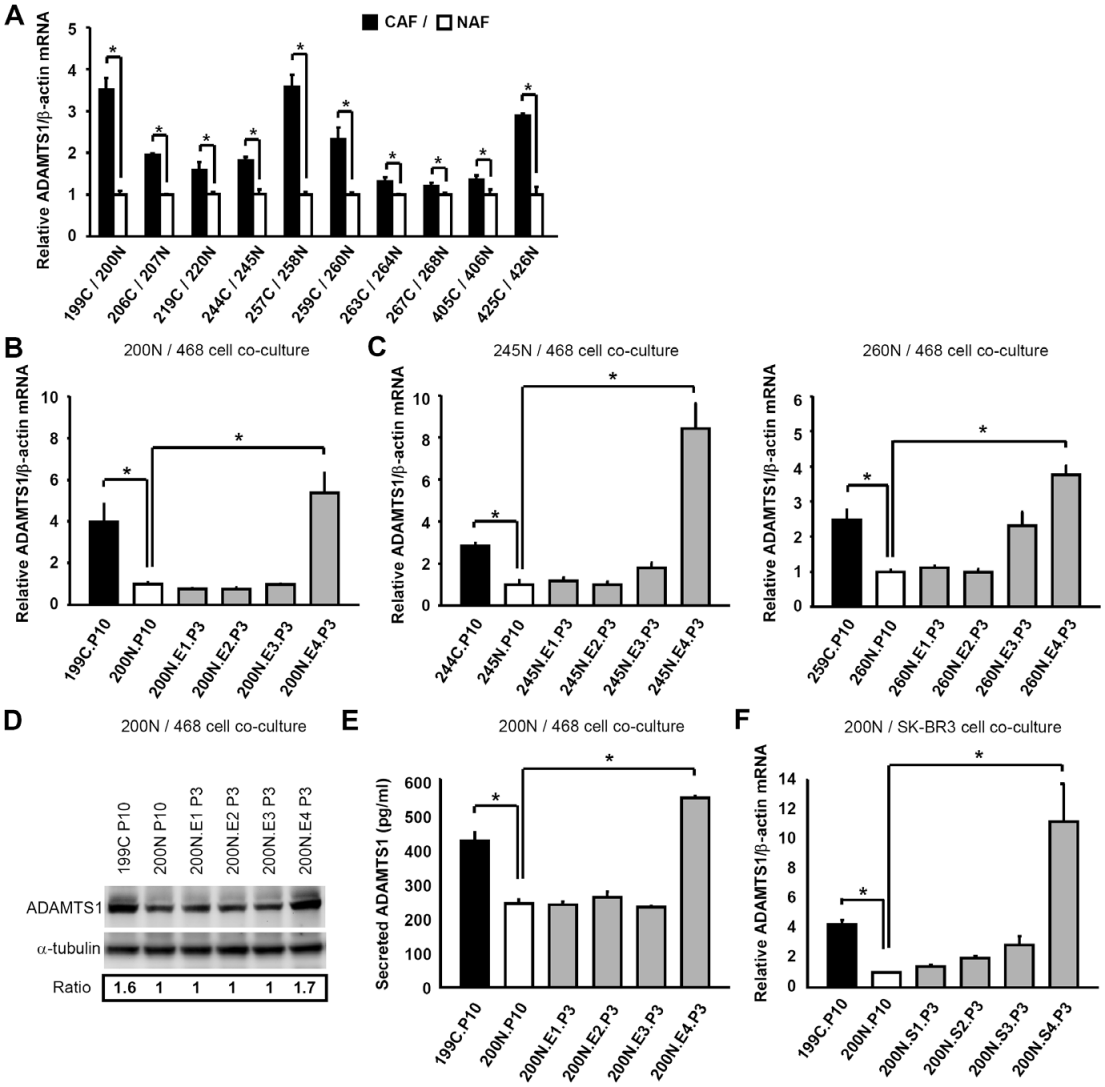
Pre-coculture with breast cancer cells increased ADAMTS1 mRNA levels in NAFs. (A–C) Quantitative real-time RT-PCR analysis revealed that ADAMTS1 mRNA levels were higher in CAFs than in the corresponding NAFs in ten breast cancer patients (A), and that ADAMTS1 mRNA levels in CAF 199C.P10 and NAF 200N.E4.P3 were higher than in NAF 200N.P10 and 200N.E1-E3.P3 (B). Similar results are shown using the pairs of CAF 244C/NAF 245N and CAF 259C/NAF 260N (C). (D) The ADAMTS1 protein level in NAF 200N.E4.P3, but not NAF 200N.E1-E3.P3, was enhanced to the similar level in CAF 199C.P10, compared to NAF 200N.P10. (E) ELISA indicated that the ADAMTS1 protein level in cultured medium derived from NAF 200N.E4.P3 was higher than NAF 200N.E1-E3.P3 and NAF 200N.P10. (F) ADAMTS1 mRNA level in SK-BR-3 cell-precocultured NAF 200N.S4.P3 was also higher than in NAF 200N.P10 and 200N.S1-S3.P3. Data are shown as mean ± SD from triplicate experiments. Statistical significance was evaluated by Student's t-test. * *P*<0.05.

Our results above suggest that paracrine signaling may be involved in the breast cancer cell invasion by the co-cultured fibroblasts. To identify the fibroblast-secreted factors that contribute to cancer cell progression, we compared gene expression profiles in CAF 199C, NAF 200N and NAF 200N.E1-E4.P3 using cDNA microarray analysis. Particular interests were paid to genes encoding secreted proteins and genes only upregulated in 199C.P10 and 200N.E4.P3 as these two cell lines show the ability to promote cancer cell invasion. Several factors including serglycin (SRGN) and ADAMTS1 (Table 1) were initially identified, but only these two genes were confirmed in 199C/200N using quantitative real-time RT-PCR analysis (Fig. 2*A* and Fig. S1). ADAMTS1 was chosen for this study due to its reported essential role in cancer metastasis [10]. Further comparative analysis of total 10 pairs of CAF/NAF from breast cancer patients revealed that the expression of ADAMTS1 in CAFs was relatively higher than in NAF (Fig. 2*A*).

**Table 1:** Relative gene expression of serglycin and ADAMTS1.

Gene Name	199C.P10	200N.P10	200N.E1.P3	200N.E2.P3	200N.E3.P3	200N.E4.P3
Serglycin	3.85	1.00	1.30	0.80	1.04	2.69
ADAMTS1	1.69	1.00	1.30	0.95	0.92	2.56

Relative gene expression levels of secreted proteins up-regulated in 199C and 200N.E4.P3. The results of cDNA microarray analysis show that serglycin and ADAMTS1 are induced in CAF 199C.P10 and MDA-MB-468 cell-precocultured NAF 200N.E4.P3, compared to NAF 200N.P10.

To conform whether ADAMTS1 expression is correlated to the invasion ability, the co-cultured NAF derivatives from Fig. 1*B* were analyzed. The co-cultured derivative 200N.E4.P3 produced high level of ADAMTS1 mRNA only after four consecutive co-cultures with MDA-MB-468 cells (Fig. 2*B*). In addition to 200N, the up-regulation of ADAMTS1 expression was also observed in other normal tissue-associated fibroblasts from two patients, 245N and 260N, after four consecutive co-cultures with MDA-MB-468 cells (Fig. 2*C*, 245N.E4.P3 and 260N.E4.P3). These results indicate that breast cancer cells are able to induce ADAMTS1 expression in the co-cultured fibroblasts. Consistently, the levels of total ADAMTS1 protein and the secreted ADAMTS1 were increased in 199C.P10 and 200N.E4.P3, but not 200N.P10 or 200N.E1-3.P3 (Fig. 2*D and 2E*). It should be noted that the ADAMTS1 doublet in Figure 2*D* likely represents differently posttranslationally modified forms of the protein. The lower band is ADAMTS1 (around 87 kD) and the upper band is the N-glycosylated form of ADAMTS1, as suggested [13]. To further confirm the induction of ADAMTS1 is not only specific to NAFs co-cultured with MDA-MB-468, NAF 200N co-cultured with another breast cancer cell line SK-BR-3 was analyzed. Similarly, ADAMTS1 mRNA level was increased in SK-BR-3 cell-precocultured NAF 200N.S4.P3, but not 200N.S1-S3.P3, compared to 200N.P10 (Fig. 2*F*). These results demonstrated that breast cancer cell-mediated ADAMTS1 induction in NAFs is likely a common event.

### ADAMTS1 secreted from fibroblasts enhanced cancer cell invasion

**Figure 3:**
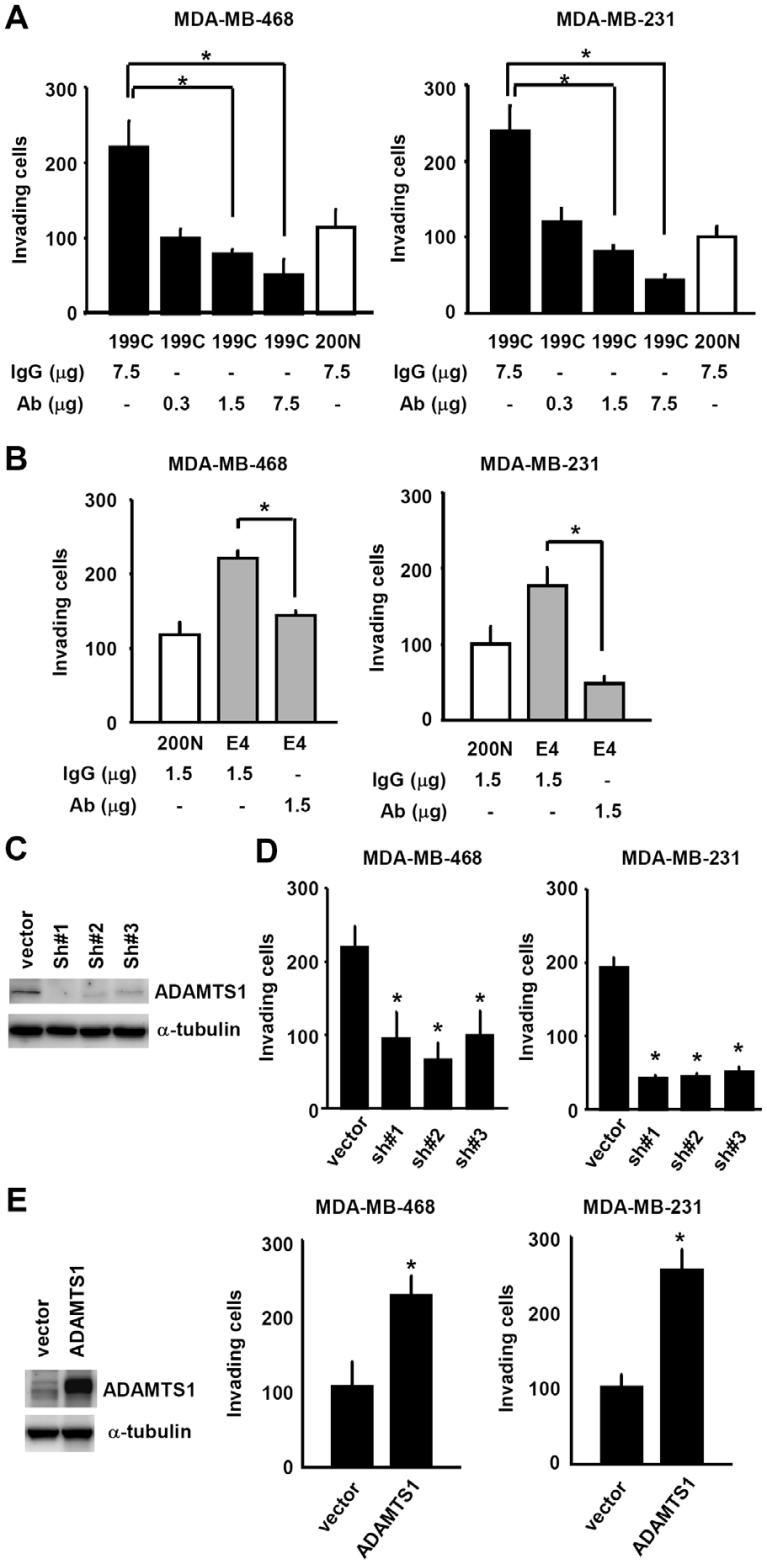
ADAMTS1 secreted from stromal fibroblasts enhanced cancer cell invasion. (A) Sequestration of the ADAMTS1 activity in the conditional medium derived from CAF 199C.P10 using an anti-ADAMTS1 antibody reduced invasion ability of MDA-MB-468 cells (left) and MDA-MB-231 cells (right) in an anti-ADAMTS1 Ab dose-dependent manner. (B) Neutralization of the ADAMTS1 activity in the conditional medium derived from NAF 200N.E4.P3 using an anti-ADAMTS1 antibody also reduced invasion ability of MDA-MB-468 cells (left) and MDA-MB-231 cells (right). (C) Western shows the efficient inhibition of ADAMTS1 protein level by three independent shRNAs. (D) The conditional medium derived from CAF 199C.P10 depleted of ADAMTS1 by shRNAs exhibited significantly lower activity for invasion of MDA-MB-468 cells (left) and MDA.MB-231 cells (right). (E) The conditional media derived from NAF 200N.P10 with exogenous ADAMTS1 expression (left, western) enhanced invasion of MDA-MB-468 cells (middle) and MDA-MB-231 cells (right). Data are shown as mean ± SD from triplicate experiments. Statistical significance was evaluated by Student's t-test. * *P*<0.05.

Subsequently we examined the cancer invasion promotion function of ADAMTS1 secreted from fibroblasts. To this purpose, ADAMTS1 Ab was added into the conditional medium derived from CAF 199C, followed by invasion assays. As shown in Fig. 3*A*, blocking of ADAMTS1 with the corresponding antibodies greatly impaired 199C's cancer invasion promotion function on both MDA-MB-468 and MDA-MB-231 cells in an ADAMTS1 Ab dose-dependent manner (Fig. 3*A*). Consistently, ADAMTS1 Ab also suppressed NAF 200N.E4.P3-mediated invasion of breast cancer cells (Fig. 3*B*). To further confirm the specificity of ADAMTS1 in the invasion promotion function, we also knocked down ADAMTS1 expression in CAF 199C using a shRNA lentiviral system (Fig. 3*C*) and found that ADAMTS1 depletion significantly reduced CAF 199C-mediated cancer invasion (Fig. 3*D*). In agreement with these results, overexpression of ADAMTS1 enhanced the invasion function of NAF 200N (Fig. 3*E*). Taken together, these experiments strongly indicate that fibroblasts promote cancer cell invasion through secreting ADAMTS1.

### ADAMTS1 expression in cancer-associated fibroblasts positively correlated with metastasis

**Figure 4:**
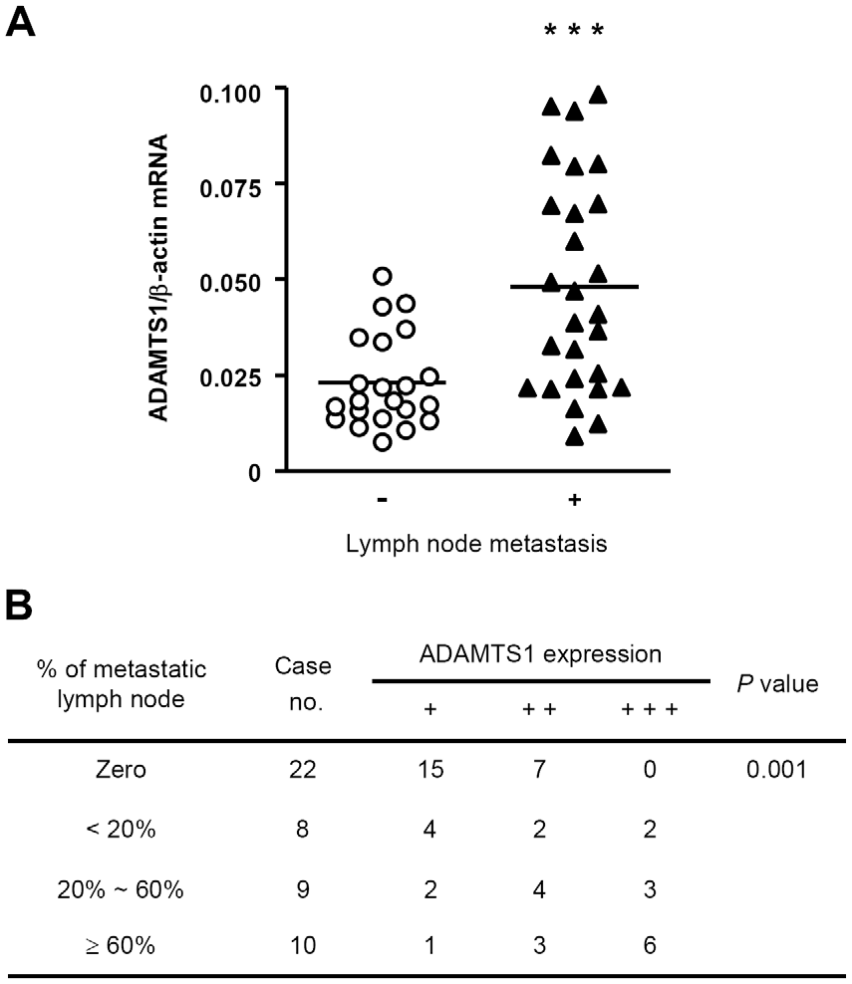
ADAMTS1 expression in CAF correlated with lymph node metastasis. (A) A scatter dot plot of ADAMTS1 mRNA levels in CAFs assessed using quantitative real-time RT-PCR analysis. The ADAMTS1 mRNA levels in CAFs of patients with lymph node metastasis (n = 27) were significantly higher than in those of patients without lymph node metastasis (n = 22) (P = 0.0003). Data are shown as mean ± SD of triplicate samples. Statistical significance was evaluated by Student's t-test. *** P<0.001. (B) The relationship between ADAMTS1 expression and lymph node metastasis was analyzed using Fisher's exact test. ADAMTS1 expression levels in CAFs derived from patients with ≥60% lymph node metastasis were significantly higher than those of patients with ≤60% lymph node metastasis or with zero lymph node metastasis (*P* = 0.001). Relative quantity (RQ) of ADAMTS1 expression compared to beta-actin expression: +, 0.001≤RQ≤0.025; ++, 0.025<RQ<0.051; +++, RQ≥0.051.

Given that cancer-associated fibroblasts secrete ADAMTS1 for cancer cell invasion, it's likely that the expression of ADAMTS1 in CAFs is physiologically relevant to breast cancer progression. To test this possibility, we analyzed ADAMTS1 mRNA of CAFs from 49 breast cancer patients and found that the higher expression of ADAMTS1 mRNA of CAFs in patients was significantly correlated with lymph node metastasis (Fig. 4*A*). Further detailed analysis by subdividing patients with degree of metastasis versus ADAMTS1 mRNA level revealed a similar significant correlation (Fig. 4*B*). These results indicated that ADAMTS1 expression in cancer-associated fibroblasts positively correlates with metastasis and thus may play an important role in breast cancer progression.

### The heritable increase of ADAMTS1 expression in cancer cell-precocultured fibroblasts correlated with reduction of ADAMTS1 promoter-associated H3K27me3 and EZH2 binding

**Figure 5:**
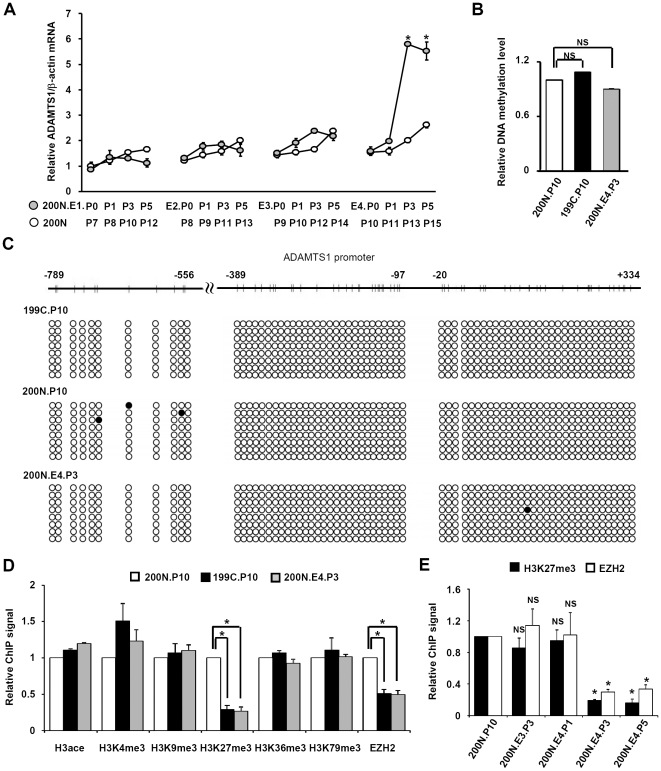
Increase of ADAMTS1 mRNA correlated with reduction of ADAMTS1 promoter-associated H3K27me3 and EZH2 binding through passages. (A) ADAMTS1 mRNA levels in indicated NAF derivatives were analyzed and shown gradually increased in NAF 200N.E4 from P0 to P3 and maintained from P3 to P5. (B) MeDIP indicates that ADAMTS1 promoter-associated DNA methylation level was similar in NAF 200N.P10, CAF 199C.P10 and NAF 200N.E4.P3 cells. (C) Bisulfite sequencing indicates that ADAMTS1 promoter in CAF 199C.P10, NAF 200N.P10 and NAF 200N.E4.P3 cells was hypomethylated. Closed circle: methylated cytosine. (D) ADAMTS1 promoter-associated H3K27me3 and EZH2 binding was decreased in CAF 199C.P10 and NAF 200N.E4.P3 cells. (B and D) MeDIP (B) or ChIP assays (D) using antibody against H3ace, H3K4me3, H3K9me3, H3K27me3, H3K36me3, H3K79me3 or EZH2 was performed in NAF 200N.P10 (white bars), CAF 199C.P10 (black bars) and NAF 200N.E4.P3 (gray bars) cells, followed by real-time PCR analysis. (E) ADAMTS1 promoter-associated H3K27me3 and EZH2 binding were decreased in NAF 200N.E4.P3 and maintained through passages to NAF 200N.E4.P5. ChIP assays using antibody against H3K27me3 (black bars) or EZH2 (white bars) were performed in cells indicated, followed by real-time PCR analysis. (B, D and E) PCR amplification was performed with DNA primers against the ADAMTS1 promoter region from −431 bp to −217 bp. Data are shown as mean ± SD from triplicate experiments. Statistical significance was evaluated by Student's t-test. * *P*<0.05. NS: No significant difference.

Finally, we investigated the mechanism by which ADAMTS1 was activated in NAF co-cultured with breast cancer cells. It should be noted that the induced expression of ADAMTS1 indeed was observed in NAF after 4 consecutive co-incubations with breast cancer cells and the following removal of breast cancer cells for 3 passages (ex, 200N.E4.P3). It's not known whether ADAMTS1 was upregulated in passages before P3 or still sustained after P3. Analysis of ADAMTS1 gene expression in 200N.E4.P0-P5 indicated that the ADAMTS1 mRNA level was gradually increased from P1 to P2, followed by a sharp induction in P3 (Fig. 5*A*). Interestingly, the ADAMTS1 mRNA level in P5 was comparable to P3, indicating that ADAMTS1 induction, once established, could be maintained (Fig. 5*A*). In contrast, the ADAMTS1 expression in 200N.E1-E3.P0-P5 or the parental 200N.P7-P15 was minimally activated (Fig. 5*A*).

Next, the potential mechanism involved in the sustained expression of ADAMTS1 in NAFs after removal of the co-cultured breast cancer cells was explored. First, we analyzed if the DNA methylation level of the ADAMTS1 promoter in 200N.E4.P3 or 199C.P10 was lower than in 200N.P10. DNA methylation correlates with gene repression and is heritable through recognition of the hemi-methylated DNA and methylation of the corresponding cytosine in the nascent DNA by the DNA methyltransferase 1 during DNA replication [14]. As shown in Fig. 5*B*, the methylated DNA immunoprecipitation (MeDIP) assays indicated that no significant change in DNA methylation was observed. The methylation of the promoter of H19, an imprinted gene permanently silenced in the paternal allele in somatic cells [15], and the promoter of ubiquitin-conjugating enzyme E2B (UBE2B), a constitutively active gene [16], was examined as positive and negative controls for MeDIP, respectively. The analysis revealed that the DNA methylation level in ADAMTS1 promoter was comparable to the low level in UBE2B gene in both 200N.P10 and 199C.P10 (Fig. S2). The bisulfite sequencing further confirmed the hypomethylation of ADAMTS1 promoter in 199C.P10, 200N.P10 and 200N.E4.P3 (Fig. 5*C*). Thus, it's unlikely that DNA methylation governs the differential expression of ADAMTS1 in cancer-associated fibroblasts.

In addition to DNA methylation, specific histone modifications also correlate with gene expression, although their heritable roles are still unsettled [17]. A panel of ADAMTS1 promoter-associated histone modifications was analyzed in 200N.P10, 199C.P10, and 200N.E4.P3 using chromatin immunoprecipitation assays with antibodies against acetylated H3, H3 tri-methylated at K4, K9, K27, K36, or K79, or the histone methyltransferase EZH2. Note that H3 acetylation, trimethylation of H3 at K4, K36 and K79 are generally associated with gene activation, while trimethylation of H3 at K9 and K27 correlates with gene repression [18], [19], [20]. As shown in Fig. 5*D*, only ADAMTS1 promoter-associated H3K27 trimethylation was reduced in 199C.P10 and 200N.E4.P3, compared to 200N.P10. Consistently, the ADAMTS1 promoter-associated binding of EZH2, the histone methyltransferase for H3K27 trimethylation, was also significantly reduced (Fig. 5*D*). The quantitative ChIP results of Fig. 5, shown by pull-down percentage, are now displayed in Fig. S3. Furthermore, the reduction of the EZH2 binding was not due to decreased EZH2 expression in 200N.E4.P3 (Fig. S4). Most importantly, the decrease of both H3K27me3 and EZH2 binding on the ADAMTS1 promoter could be sustained through passages to 200N.E4.P5 (Fig. 5*E*). These results indicated that the heritable increase of ADAMTS1 expression in fibroblasts pre-cocultured with cancer cells is likely mediated by the reduction of ADAMTS1 promoter-associated EZH2 binding and the resulting loss of H3K27me3.

## Discussion

The current report demonstrates that cancer-associated fibroblasts (CAFs) secreted ADAMTS1 to promote cancer cell invasion (Fig. 3). We further show that the level of the CAF-secreted ADAMTS1 significantly correlates with lymph node metastasis of breast cancer patients (Fig. 4). Indeed, a previous study has revealed a role of cancer cell-expressed ADAMTS1 in metastasis with a mechanism involving activation of epidermal growth factor receptor and ErbB-2 and shedding of amphiregulin and heparin-bound epidermal growth factor precursors [10]. Interestingly, we found that both the mRNA and protein levels of ADAMTS1 in CAFs are much higher than those in cancer cells such as MDA-MB-468 and MDA-MB-231 (Fig. S5). Given that CAF-secreted ADAMTS1 exhibits promotion ability for cancer invasion (Fig. 3), it's highly possible that ADAMTS1 secreted from CAFs contributes more than that from cancer cells to facilitate cancer invasion.

Our study further points out that the activation of ADAMTS1 in CAFs can be recapitulated in normal tissue-associated fibroblasts (NAFs) after their co-culture with breast cancer cells (Fig. 2), and that ADAMTS1 stimulation in cancer cell-cocultured NAFs can be sustained after the removal of the co-cultured cancer cells (Fig. 2). The latter observation is particularly intriguing. To explore the underlying mechanism, we investigated two most well studied causes for gene repression: DNA methylation and histone methylation. It was found that the sustained ADAMTS1 upregulation in NAFs precocultured with cancer cells correlates with an epigenetic mechanism involving the loss of ADAMTS1 promoter-associated EZH2 binding and the corresponding H3K27 trimethylation (Fig. 5). In contrast, DNA methylation does not seem to participate in the event (Fig. 5B, 5C, and S2). Although ADAMTS1 promoter has been shown to contain CpG islands which are hypermethylated in colorectal cancer [21], our bisulfite sequencing and MeDIP results indicate that ADAMTS1 promoter is indeed hypomethylated in breast cancer-associated fibroblasts, normal breast tissue-associated fibroblasts or NAFs co-cultured with breast cancer cells (Fig. 5B, 5C and S2). Thus, it's very likely that the change in H3K27 trimethylation, rather than DNA methylation, governs this epigenetic memory, which contributes to the sustained expression of ADAMTS1 in cancer cell-precocultured NAFs. These results are also consistent with previous observations in which DNA methylation and H3K27 methylation, though both are correlated with gene repression, separately control independent subsets of genes [22], [23], [24], [25]. Since histone methylation is a balanced reaction carried out by histone methyltransferases and demethylases, the possibility that H3K27 demethylases such as UTX and JMJD3 [26], [27] may be used to remove the methyl marks from ADAMTS1 promoter cannot be excluded.

Importantly, we found that H3K27me3 is also reduced in the promoter of serglycin, a proteoglycan [28], in 199C.P10 and 200N.E4.P3 where serglycin mRNA level is increased (Table 1, Fig. S1 and S6). This suggests that loss of the transcriptional repression mark H3K27me3 is likely a common mechanism to derepress genes essential for cancer promotion function of cancer cell-associated fibroblasts.

How ADAMTS1 expression is induced following co-culture with breast cancer cells remains to be resolved. The mechanism, which prevents EZH2 from binding to ADAMTS1 promoter in cancer cell-precocultured NAFs, is currently under investigation. A previous report has shown that overexpression of Akt in breast cancer cells renders EZH2 phosphorylated at Ser 21 and results in the loss of EZH2 binding to its target promoters [29]. Consistently, our preliminary data that LY294002, an inhibitor of PI3K-Akt signaling pathway, significantly diminished ADAMTS1 activation in cancer cell-precocultured NAF, suggesting that PI3K-Akt signaling may mediate the initial step to down-regulate the binding of EZH2 to ADAMTS1 promoter (data not shown). Furthermore, it remains obscure what kind of factors secreted from breast cancer cells is responsible for “educating” NAFs either directly or indirectly. This process is of great interest for further pursuing. Given the increased expression of ADAMTS1, the altered histone methylation at the ADAMTS1 promoter is not particularly surprising. Indeed, the most intriguing question is how the loss of EZH2 binding to ADAMTS1 promoter is sustained in cancer cell-cocultured NAFs after the removal of cancer cells. Currently, there is little clue to address this issue. Nevertheless, the system described here provides a potential venue to demystify how epigenetic change becomes inheritable.

## Materials and Methods

### Ethics statement

All human specimens were encoded to protect patient confidentiality and processed under protocols approved by the Institutional Reviews Board of Human Subjects Research Ethics Committee of Academia Sinica (AS-IRB02-98042) and National Taiwan University Hospital (#200902001R), Taipei, Taiwan. Breast cancer tissues and its relative normal counterparts were obtained from patients who underwent surgery at National Taiwan University Hospital. Signed consent for the studies was obtained from all the patients.

### Clinical specimens and cell cultures

Human breast cancer tissues and its relative normal counterparts were minced to 2–3 mm^3^ cubes and attached onto the culture dishes for fibroblast culture. Primary fibroblasts isolated from clinical specimens were maintained in Dulbecco's Modified Eagle's Medium/F-12 Nutrient Mixture (DMEM/F-12) (Invitrogen) supplemented with 10% fetal bovine serum (Industrial Biological), 0.1 mM non-essential amino acids and 1 mM sodium pyruvate (Invitrogen). Breast cancer cell lines, MDA-MB-468, MDA-MB-231 and SK-BR-3, were cultured in DMEM/F-12, supplemented with 10% fetal bovine serum.

### Plasmid DNA and lentiviral infection

Lentivirus-based shRNA plasmids for knocking down ADAMTS1 expression were provided by National RNAi Core Facility, Taiwan. The target sequences were 5′-CCA GCA TTC GTA ATT CAG TTA-3′ (sh#1), 5′-CCA CAG GAA CTG GAA GCA TAA-3′ (sh#2) and 5′-CCA CCT TAG AGC AAG ACA TTA-3′ (sh#3). Viral infection was performed following the procedures provided by National RNAi Core Facility. ADAMTS1 cDNA was derived and amplified from Origene's human full-length cDNA collection using the following primers: 5′-CGA TCT CGA GAT GCA GCG AGC TGT GCC CG-3′ and 5′-CGA TTC TAG ATT AAC TGC ATT CTG CCA TTG TG-3′. The amplified DNA fragment was subcloned into pLVX-IRES-Neo vector using restriction endonuclease XhoI and XbaI for expression through a Lenti-X expression system (Clontech).

### Co-culture of fibroblasts with cancer cells

Co-culture of fibroblasts with cancer cells was performed using a transwell system as described by Tyan *et al*. [4]. In the co-culture system, 8×10^4^ NAF 200N.P7 cells were grown in the bottom of a 6-well plate in 2.5 ml of DMEM/F-12 with 10% fetal bovine serum and 1×10^5^ MDA-MB-468 cells were seeded onto the 0.4-µm polyester membrane of a transwell insert (Corning) in 1.5 ml of the same medium. NAF 200N.E1.P0 was derived after incubation of the co-cultured cells for 78 h in a humidified incubator with 5% CO_2_ at 37°C. NAF 200N.E1.P0 fibroblasts were subcultured and grown for generation of passages P1, P2 and P3 without MDA-MB-468 cell co-culture; others were subcultured and continued to co-culture with MDA-MB-468 cells for 78 h to generate NAF 200N.E2.P0. NAF 200N.E3.P0 and NAF 200N.E4.P0 and their P1 to P3 derivatives were obtained following the same procedure described above.

### Invasion assay

10^4^ of MDA-MB-468 or MDA-MB-231 cells were suspended in 100 µl of the 24 h-cultured media collected from indicated fibroblast derivatives at 100% confluency. The mixtures were subsequently added onto the upper chamber of a transwell apparatus containing an insert with a proprietary light-opague PET membrane of 8-µm pore size (Falcon #351152) and coated with 15 µl of 30 µg matrigel matrix (BD #354234). The lower chamber was added with 500 µl of medium containing 10% FBS. The cells were incubated for 16 h or 36 h for MDA-MB-231 or MDA-MB-468 cells, respectively. The invading cells on the lower surface of membrane were fixed with methanol for 20 min, washed twice with PBS for 5 min, stained with DAPI (1∶10000) for 5 min, and examined and counted with fluorescence microscopy.

For the purpose of neutralizing ADAMTS1 activity, 0.3–7.5 µg of mouse monoclonal anti-human ADAMTS1 antibody (R&D Systems) or mouse IgG_2B_ (R&D Systems) was added to 100 µl of 100% confluent fibroblast-cultured medium. The medium was then mixed with MDA-MB-468 or MDA-MB-231 cells before the invasion assays were performed.

### Quantitative real-time RT-PCR

Total RNAs from fibroblasts were isolated using TRI reagent (Ambion). One µg of purified RNA was subjected to cDNA synthesis by Superscript II reverse transcriptase (Invitrogen) in 20 µl of reaction buffer according to the manufacturer's protocol, followed by real-time PCR analysis using the ABI PRISM 7000 sequence detection system with SYBER Green method (Applied Biosystems) according to the manufacturer's instruction. The primers used were: ADAMTS1: 5′-ACG AGT GCG CTA CAG ATC CT-3′ and 5′-CAG CGT ACT TGG GAA TCC AT-3′; SRGN: 5′-CGC TGC AAT CCA GAC AGT AA-3′ and 5′-CCT GGA TTC TCG TCT TTG GA-3′; the internal control, beta-actin: 5′-ATC TGG CAC CAC ACC TTC TAC A-3′ and 5′-TCA CCG GAG TCC ATC ACG AT-3′. The amplification mixture contained 1 µl of 5× diluted reverse transcription product, 200 nM of each primer, 250 nM probe, and 12.5 µl of 2× SYBER Green PCR master mix (Applied Biosystems) in a total of 20-µl reaction volume. The thermal conditions were: 2 min at 50°C and 10 min at 95°C, followed by 40 cycles at 95°C for 15 sec and 55°C for 1 min. The relative quantity of mRNA was estimated by using a standard curve created by serial dilution of the reverse transcription products from NAFs.

### Western blotting analysis

Fibroblasts were grown to 100% confluency and replaced with fresh medium one day before protein extraction with RIPA buffer containing 50 mM Tris-HCl (pH 7.4), 150 mM NaCl, 1% IGEPAL CA-630, 2 mM EDTA and protease inhibitors with thorough homogenization. Lysates were centrifuged and the supernatant with 30 µg of protein content was resolved by SDS-8% PAGE, followed by protein transfer to the PVDF membrane and immunoblotting of the membrane with mouse monoclonal anti-human ADAMTS1 (GeneTex), anti-alpha tubulin (GeneTex) or rabbit polyclonal anti-EZH2 (Diagenode) antibody. The secondary antibody used was horseradish peroxidase (HRP)-conjugated horse-anti-mouse IgG (Cell Signaling) or HRP-conjugated donkey-anti-rabbit IgG (GE Healthcare). Membranes were developed by reacting with chemiluminescent HRP substrate and exposed to BioSpectrumAC imaging system (Ultra-Violet Products).

### Enzyme-linked immunosorbent assay (ELISA) to quantify the secreted ADAMTS1

Fibroblasts were grown to 100% confluency and replaced with fresh medium one day before the assay. The cultured medium was centrifuged at 300×g for 5 min. 100 µl of the supernatant was subjected to enzyme-linked immunosorbent assay for ADAMTS1 (Uscn Life Science) following the manufacturer's instruction.

### Methylated DNA immunoprecipitation (MeDIP) assay

Genomic DNA was prepared using QIAamp DNA mini kit (Qiagen) and sonicated using Bioruptor sonication device (Diagenode) to produce random fragments ranging in mean size from 300 to 1,000 bp. 5 µg of fragmented DNA was denatured for 10 min at 95°C and immunoprecipitated overnight at 4°C with 5 µl of 5-methylcytidine antibody (Eurogentec) in a final volume of 500 ml IP buffer [10 mM sodium phosphate (pH 7.0), 140 mM NaCl, 0.05% Triton X-100]. The mixture was incubated with 30 µl magnetic beads (Millipore) for another 4 h at 4°C and washed three times with 1 ml of IP buffer. Beads were resuspended with 250 µl digestion buffer (50 mM Tris pH 8.0, 10 mM EDTA, 0.5% SDS) containing 0.4 mg/ml protease K and shaken overnight at 56°C. DNA was extracted using QIAquick PCR purification kit (Qiagen) and subjected to real-time PCR analysis using the Roche Light Cycler 480 real-time PCR system with SYBER Green method (Roche) according to the manufacturer's instruction. Primers for ADAMTS1 promoter from −431∼−217 bp were 5′-CCA GGA TAG GGA AAT GTT GA-3′ and 5′-TGT GAC CAG CAC TTT GTA CT-3′.

### Bisulfite sequencing analysis

Genomic DNA was prepared using Genomic DNA mini kit (Geneaid) and subjected to bisulfite CT conversion reaction using EZ DNA methylation-Gold kit (ZYMO research). The promoter and 5′-untranslated region of ADAMTS1 was divided into four fragments, which were amplified from the bisulfite reaction mixture using the following primers: for nt −813∼−501 region: 5′-TTT TTA TGT TTT AGG GAA TTG-3′ and 5′-AAA AAT ACC TCA CCC CAC TT-3′; for nt −514∼−219 region: 5′-GGT GAG GTA TTT TTT TAG AA-3′ and 5′-TAA CCA ACA CTT TAT ACT AC-3′ ; for nt -268∼+51 region: 5′-AGG GAG AGT TTT TTT TTT GGA-3′ and 5′-CAC CCT AAC TTT ACA ATA AC-3′ ; for nt +64∼+370 region: 5′-GAG AGG GGA GAG TTT TGA GTA GAG T-3′ and 5′-ACT CTA AAT TAT TAA AAT TAA CAA TTT CTA-3′. The amplified fragments were subcloned into pCR2.1-TOPO vector (Invitrogen) for sequencing analysis.

### Chromatin immunoprecipitation (ChIP)

ChIP assays were performed as described by Hsu *et al*. [30] with modifications. The antibodies used for immunoprecipitation were H3ace (Millipore #06-599), H3K4me3 (Abcam #ab1012), H3K9me3 (Abcam #ab8898), H3K27me3 (Abcam #ab6002), H3K36me3 (Abcam #ab9050) and H3K79me3 (Abcam #ab2621). The immunoprecipitated DNA fragments were extracted using QIAquick PCR purification kit (Qiagen) and subjected to real-time PCR analysis using the Roche Light Cycler 480 real-time PCR system with SYBER Green method (Roche). The amplifications with DNA primers against the ADAMTS1and SRGN promoter region were performed in a reaction volume of 20 µl containing 2 µl of immunoprecipitated material. Primers for ADAMTS1 promoter from −431 to −217 bp were 5′-CCA GGA TAG GGA AAT GTT GA-3′ and 5′-TGT GAC CAG CAC TTT GTA CT-3′. Primers for SRGN promoter from −149 to −19 bp were 5′-GGA GTC CAG TAC AGT TTC ATA AT-3′ and 5′-TGC CCA GAA CAC ACG TCA-3′.

## Supporting Information

Figure S1 **Quantitative real-time RT-PCR analysis shows that serglycin, which was highly expressed in CAF 199C.P10 compared to NAF 200N.P10, was induced in MDA-MB-468 cell-precocultured NAF 200N.E4.P3, but not in 200N.E1-E3.P3.**
(TIF)

Figure S2 **Quality controls for methylated DNA immunoprecipitation (MeDIP).** MeDIP was applied with PCR primers to amplify the promoter region of ADAMTS1, H19, an imprinted gene permanently silenced in somatic cells, or ubiquitin-conjugating enzyme E2B (UBE2B), a constitutively active gene in NAF 200N.P10 (white bars) and CAF 199C.P10 (black bars).
(TIF)

Figure S3 **The quantitative ChIP results of** **Fig. 5** **, shown by pull-down percentage.**
(TIF)

Figure S4 **The protein level of EZH2 is approximately equal in NAF 200N.P10 and NAF 200N.E4.P3 cells.** Western analysis was performed using antibody against EZH2 or alpha-tubulin.
(TIF)

Figure S5 **ADAMTS1 mRNA (A) and protein (B) levels are high in CAF 199C and NAF 200N, compared to MDA-MB-468 cells and MDA-MB-231 cells.**
(TIF)

Figure S6 **Serglycin promoter-associated H3K27me3, but not H3ace, is reduced in CAF 199C.P10 and NAF 200N.E4.P3, compared to NAF 200N.P10 cells.** Chromatin-immunoprcipitation (ChIP) assays using antibody against H3ace or H3K27me3 were performed in indicated cells, followed by PCR amplification with DNA primers against the serglycin promoter region from −149 bp to −19 bp. Data are shown as mean ± SD from triplicate experiments. Statistical significance was evaluated by Student's t-test. * *P*<0.05.
(TIF)
