# Supplementary figures and images for: Breast Cancer Cells Induce Stromal Fibroblasts to Secrete ADAMTS1 for Cancer Invasion through an Epigenetic Change
Source: PLoS One. 2012 Apr 13;7(4):e35128. doi: 10.1371/journal.pone.0035128 (PMC3325931; doi:10.1371/journal.pone.0035128)

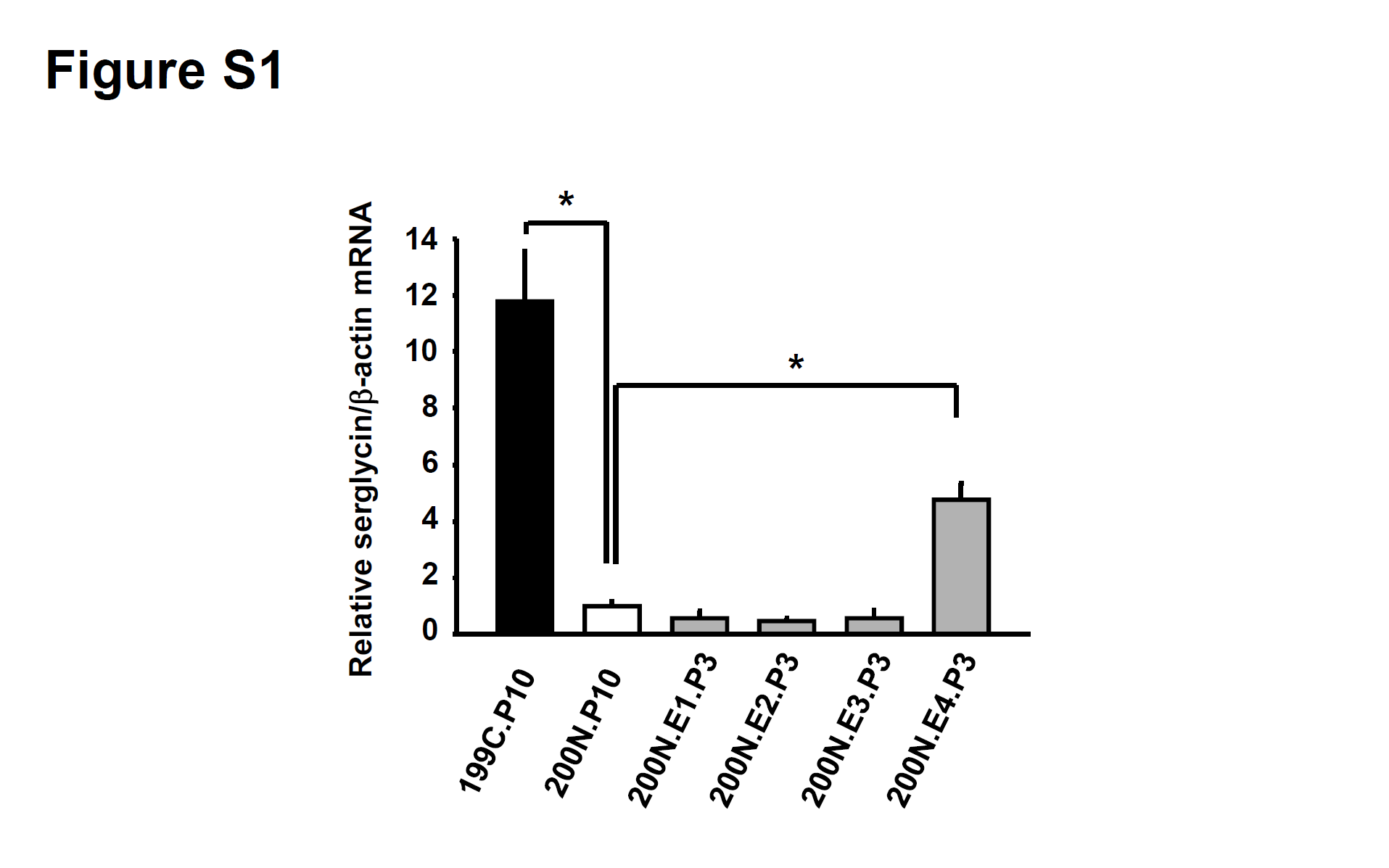

Supplement: Figure S1 — Quantitative real-time RT-PCR analysis shows that serglycin, which was highly expressed in CAF 199C.P10 compared to NAF 200N.P10, was induced in MDA-MB-468 cell-precocultured NAF 200N.E4.P3, but not in 200N.E1-E3.P3. (TIF) [file pone.0035128.s001.tif]

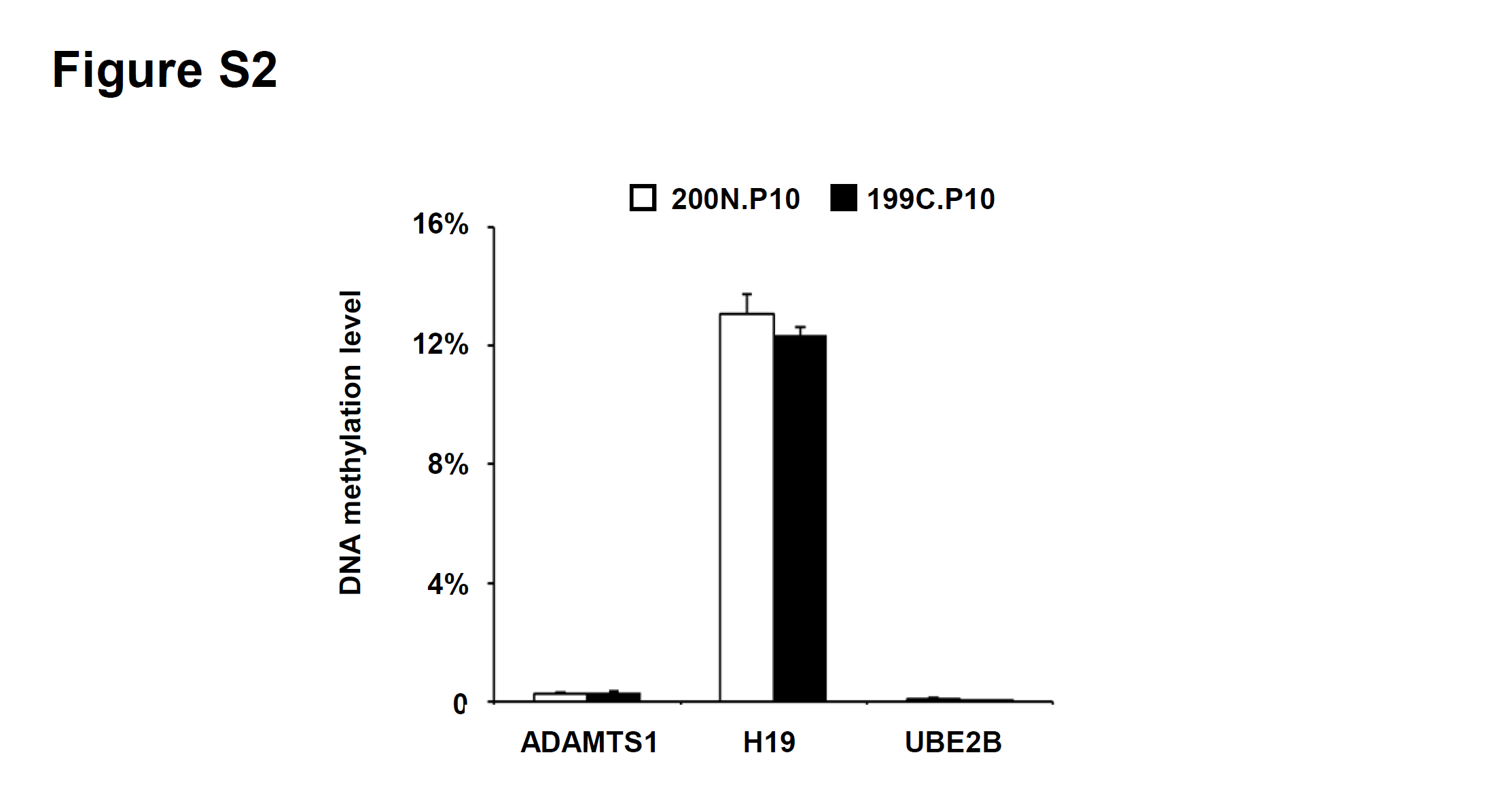

Supplement: Figure S2 — Quality controls for methylated DNA immunoprecipitation (MeDIP). MeDIP was applied with PCR primers to amplify the promoter region of ADAMTS1, H19, an imprinted gene permanently silenced in somatic cells, or ubiquitin-conjugating enzyme E2B (UBE2B), a constitutively active gene in NAF 200N.P10 (white bars) and CAF 199C.P10 (black bars). (TIF) [file pone.0035128.s002.tif]

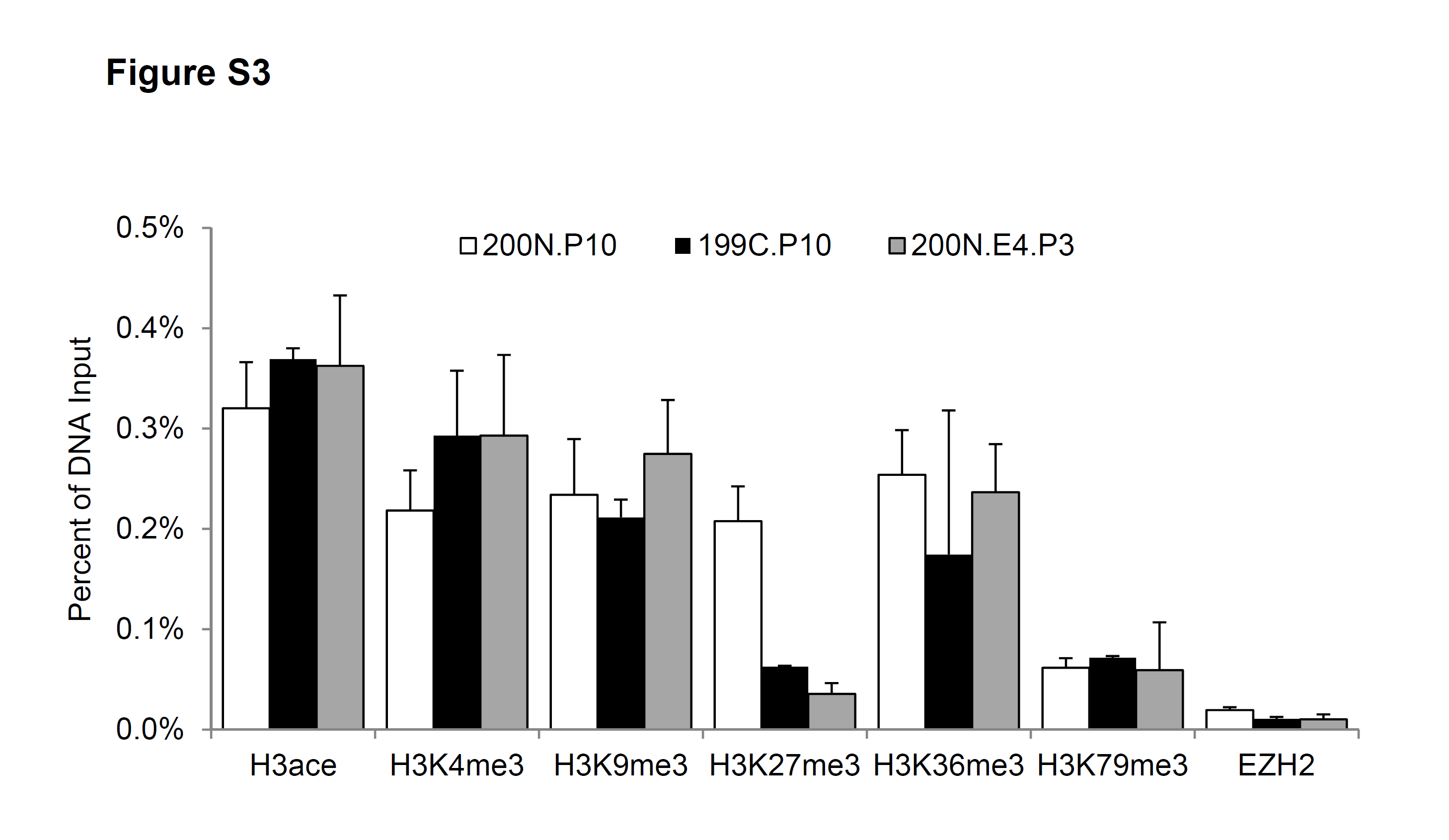

Supplement: Figure S3 — The quantitative ChIP results of Fig. 5 , shown by pull-down percentage. (TIF) [file pone.0035128.s003.tif]

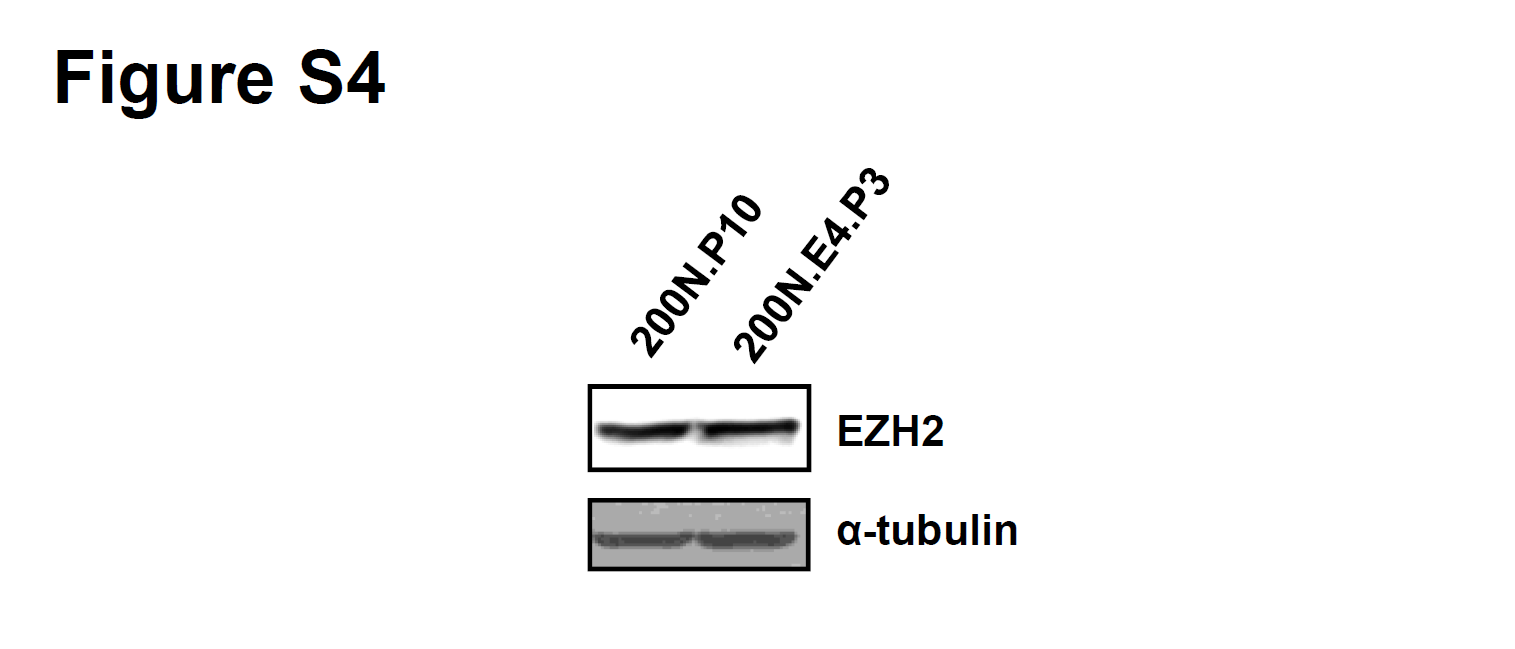

Supplement: Figure S4 — The protein level of EZH2 is approximately equal in NAF 200N.P10 and NAF 200N.E4.P3 cells. Western analysis was performed using antibody against EZH2 or alpha-tubulin. (TIF) [file pone.0035128.s004.tif]

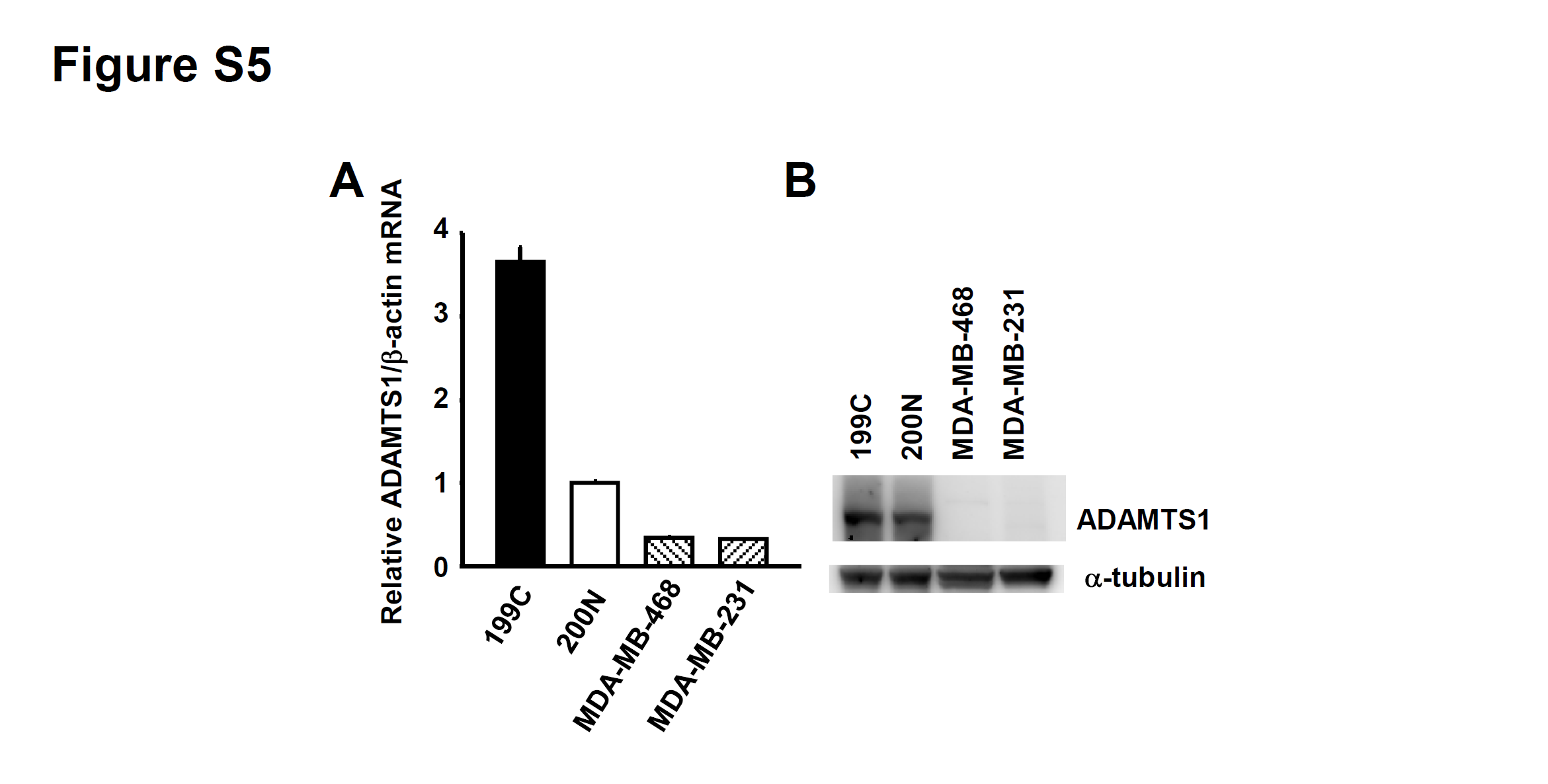

Supplement: Figure S5 — ADAMTS1 mRNA (A) and protein (B) levels are high in CAF 199C and NAF 200N, compared to MDA-MB-468 cells and MDA-MB-231 cells. (TIF) [file pone.0035128.s005.tif]

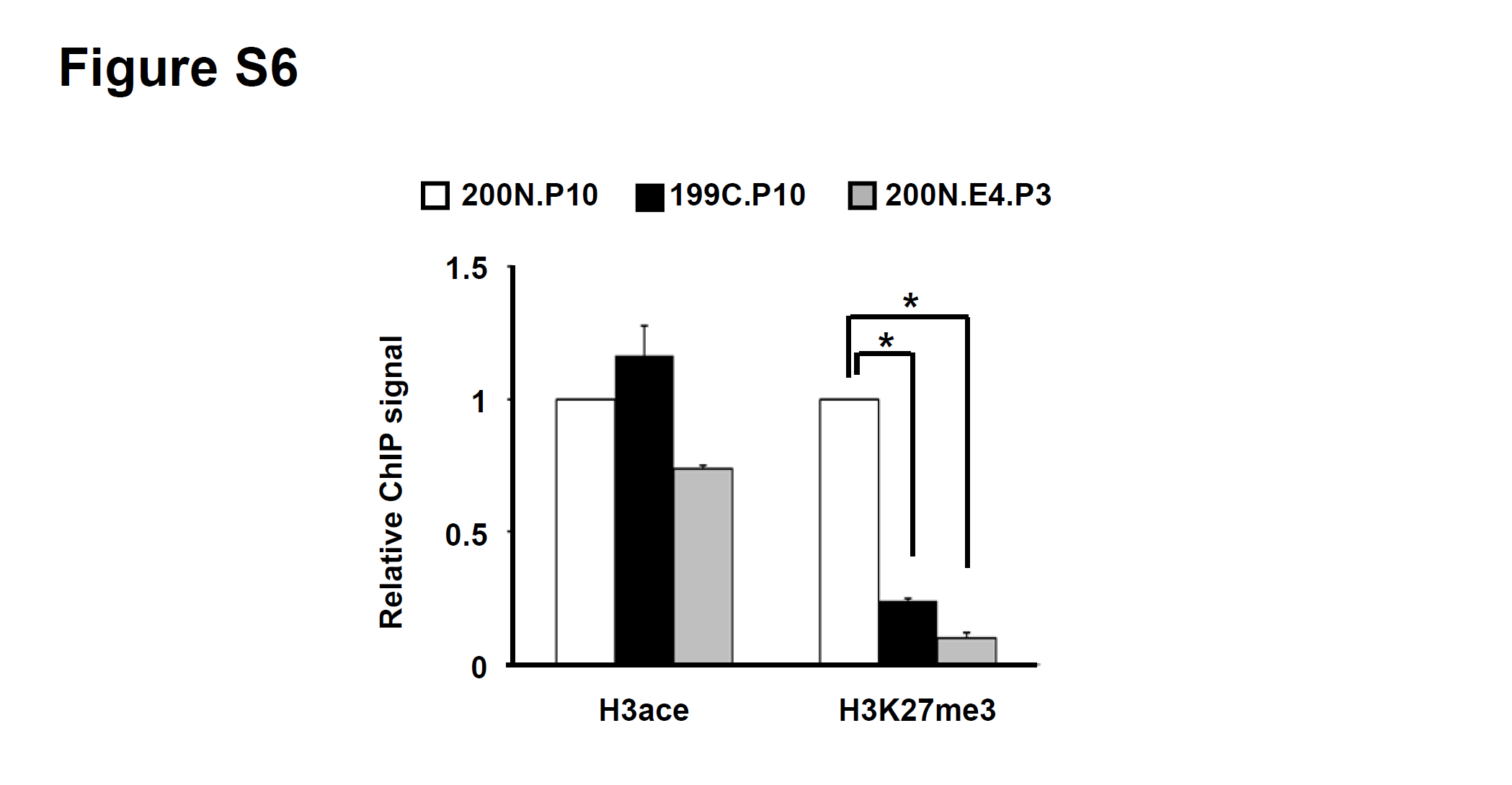

Supplement: Figure S6 — Serglycin promoter-associated H3K27me3, but not H3ace, is reduced in CAF 199C.P10 and NAF 200N.E4.P3, compared to NAF 200N.P10 cells. Chromatin-immunoprcipitation (ChIP) assays using antibody against H3ace or H3K27me3 were performed in indicated cells, followed by PCR amplification with DNA primers against the serglycin promoter region from −149 bp to −19 bp. Data are shown as mean ± SD from triplicate experiments. Statistical significance was evaluated by Student's t-test. * P<0.05. (TIF) [file pone.0035128.s006.tif]
